# Legal Phraseology

**Published:** 1885-12

**Authors:** 


### LEGAL PHRASEOLOGY.

If a man would, according to law, give to another an orange, instead of saying, "I give you that orange"—which one would think would be what is called in legal phraseology "an absolute conveyance of all right and title therein"—the phrase would run thus: "I give you all and singular my estate and interest, right, title, and claim, and advantage of, and in that orange, with all its rind, skin, juice, pulp, and pips, and all right and advantage therein, with full power to bite, cut, suck, and otherwise eat the same, or give the same away, as fully and effectually as I, said A. B., am now entitled, to bite, cut, suck or otherwise eat the same orange, or give the same away with or without its rind, juice, pulp and pips, anything heretofore or hereafter, or in any other deeds, instrument or instruments of what nature or kind soever to the contrary in any wise notwithstanding."

